# Pathological Complete Response in Rectal Cancer Patients: A Correlation Between Pathological and Clinical Stage and Oncological Outcome

**DOI:** 10.3390/cancers18020223

**Published:** 2026-01-11

**Authors:** Ana Grigoraș, Dragoș-Viorel Scripcariu, Ionuț Huțanu, Bogdan Filip, Mihaela-Mădălina Gavrilescu, Maria-Gabriela Aniței, Gheorghe Bălan, Viorel Scripcariu

**Affiliations:** 1 Faculty of Medicine, Grigore T. Popa University of Medicine and Pharmacy, 700115 Iasi, Romania; grigoras.ana@umfiasi.ro (A.G.); ionut.hutanu@umfiasi.ro (I.H.); bogdan.filip@umfiasi.ro (B.F.); gavrilescu.mihaela@umfiasi.ro (M.-M.G.); maria-gabriela.anitei@umfiasi.ro (M.-G.A.); viorel.scripcariu@umfiasi.ro (V.S.); 2 First Oncological Surgery Unit, Regional Institute of Oncology, 700483 Iași, Romania; 3 Institute of Gastroenterology and Hepatology Iași, 700111 Iași, Romania

**Keywords:** neoadjuvant treatment, pathological complete response, rectal cancer, response to treatment

## Abstract

Introduction: In rectal cancer, the choice of treatment strategy depends on the tumor stage and the response to neoadjuvant therapy. Accurate assessment of tumor regression through magnetic resonance imaging (MRI) may help to guide personalized approaches, including delayed or nonoperative management. This study aimed to assess the correlations between pathological complete response (pCR) and clinical staging before and after neoadjuvant treatment in rectal cancer patients. Methods: We conducted a retrospective analysis of rectal cancer patients treated with neoadjuvant therapy followed by radical resection in our oncological surgery department between July 2012 and December 2024. Clinical staging and tumor response were assessed using MRI, focusing on T- and N-stage evaluation. Pathological complete response (pCR) was defined as the absence of tumor cells on histopathological examination. Associations between pCR and clinical variables were explored. Results: Out of a total of 1693 rectal cancer patients, 783 (46.25%) received neoadjuvant therapy, with 62 patients (7.92%) presenting pCR. The majority had tumor stage cT3 (*n* = 45, 72.6%) and lymph node stage cN2b (*n* = 25, 40.3%) before treatment. Post-treatment MRI showed complete tumor response (T0) in 20 patients (32.3%) and nodal downstaging to N0 in 34 patients (54.8%). MRI provided imaging findings that indicate a limited correlation between clinical assessment of tumor response and pathological outcome. Six patients (9.6%) developed distant metastases, and there were no local recurrences. Conclusions: While MRI provides valuable preoperative information, its accuracy in predicting pCR remains limited. Achieving pCR is a favorable prognostic indicator, but it does not eliminate the risk of distant metastasis; therefore, continued surveillance and individualized management strategies remain essential to optimize outcomes in rectal cancer patients.

## Simple Summary

Rectal cancer is often treated with chemotherapy and radiation before surgery, and healthcare professionals rely on magnetic resonance imaging to estimate how well the tumor has responded to neoadjuvant treatment. Some patients show a complete pathological disappearance of cancer cells after treatment, which is linked to better outcomes and may allow for more personalized care, including delaying or even avoiding surgery. This study examined how well magnetic resonance imaging predicts a complete response by comparing imaging results before and after treatment with the final findings after surgery. We found that magnetic resonance imaging often underestimated or overestimated the true amount of remaining cancer. Although patients with a complete response had good outcomes, some still developed disease in other parts of the body. These findings highlight the need for careful follow-up and individualized treatment plans, as well as improved tools for accurately assessing tumor response.

## 1. Introduction

For a substantial time, the standard of care for locally advanced rectal cancer (LARC) was neoadjuvant long-course chemoradiotherapy followed by rectal resection with total mesorectal excision (TME) and postoperative adjuvant chemotherapy [1]. Gradually, the treatment algorithm has increased in complexity, aiming towards improved surgical and oncological outcomes and a higher quality of life. Available options include total neoadjuvant therapy (TNT, adding systemic chemotherapy prior to surgery) [2] and nonoperative management strategies, which require high compliance with rigorous follow-up protocols for the timely detection of tumor regrowth [3,4].

Advances in neoadjuvant treatment (NAT) have resulted in better tumor response and a higher rate of complete response; the criteria for NAT lead to a thorough selection of patients based on vigorous MRI criteria, such as tumor location, grade of perirectal infiltration, status of the mesorectal fascia, lymph node involvement, and the presence of extramural vascular invasion [3,5]. Complete pathological response (pCR) is defined as the absence of residual tumor cells at the primary tumor site and in the mesorectal lymph nodes, according to the American Joint Committee on Cancer (AJCC) Cancer Staging Manual, 8th Edition and the College of American Pathologists [6,7]. An accurate evaluation of the tumor response after NAT provides the opportunity for identifying clinical complete response (cCR), defined as the absence of detectable macroscopic tumor by clinical means after neoadjuvant treatment [4,8], which is assessed through proctologic and endoscopic examination—where the findings may include a flat white scar, small telangiectasias, no ulcer, and no nodularity [9]—and through MRI with ymrT0 ymrN0: scar not thicker than the rectal wall, only dark T2 signal [4], or the absence of a contrast-enhancing lesion; an MRI TRG between 3 and 5 [10]; and no visible lymph nodes or restricted diffusion.

The watch-and-wait approach was developed as a nonoperative strategy that can be applied in selected cases [11]; although in patients with cCR, it provides the benefits of organ preservation, lack of postoperative complications, and oncological outcomes similar to radical surgical treatment [10], there still exists a reluctance to use it. Nonoperative management means relying on indirect methods to assess a malignancy that can only be certainly diagnosed and characterized through anatomopathological examination.

In the present study, we aim to evaluate whether patients with pCR are clinically assessed as cCR prior to surgery through MR imaging, and to evaluate local regrowth and distant metastasis.

## 2. Materials and Methods

The medical charts of all patients with rectal cancer admitted to our surgical oncology unit between July 2012 and December 2024 were retrospectively reviewed. The inclusion criteria for the study were as follows: histologically confirmed rectal cancer, completion of neoadjuvant therapy independent of the chosen regimen, subsequent radical surgical resection, and achievement of complete pathological response (pCR). The exclusion criteria consisted of conservative treatment, palliative surgical treatment, patients who did not undergo NAT, and lack of pCR. The observed complete pathological response was incidental, as neoadjuvant chemotherapy was not planned or administered with the intention of achieving this outcome.

Digital rectal examination was performed at the initial clinical examination and before surgical treatment to assess response to NAT and to establish the appropriate surgical technique. Diagnosis of rectal cancer was confirmed by biopsy samples obtained during colonoscopy. Local staging was primarily performed using pelvic magnetic resonance imaging (MRI), with imaging interpretation standardized across the study period. Computed tomography (CT) was used in selected cases where MRI was contraindicated due to a patient’s obesity or medical implants. All cases were staged according to the 8th edition of the American Joint Committee on Cancer (AJCC) TNM classification. Endoscopic ultrasound was not available in our institute for systematic evaluation of tumor staging; thus, it was not performed.

Neoadjuvant therapy (i.e., radiotherapy and chemotherapy regimens) was recommended based on current clinical guidelines and tailored to individual patient profiles, following multidisciplinary team (MDT) discussions. Treatment consisted of either long-course chemoradiotherapy (LCRT)—external beam radiotherapy (50.4–54 Gy) delivered in daily fractions of 1.8 Gy over 28–30 days, with concomitant fluoropirimidine-based chemotherapy (capecitabine or 5-fluoro-uracil), the standard treatment that was applied in our institute—or short-course radiotherapy (SCRT): 25 Gy delivered in five consecutive daily fractions, only recommended in the first few years in our study period. In selected cases, induction or consolidation neoadjuvant chemotherapy (2–4 cycles of 5-FU and oxaliplatin—CAPOX regimen) was added.

Clinical response to neoadjuvant therapy was typically assessed via pelvic MRI 8 to 12 weeks after the completion of treatment. Imaging interpretation and clinical staging were consistently performed by the same medical team, consisting of a group of four radiologists specializing in the assessment of rectal cancer, who had access to patients’ clinical background. Pelvic MRI interpretation focused on T2-weighted images and aspects of contrast-enhanced lesions for appreciating clinical response; diffusion-weighted imaging (DWI) has only become available in recent years and was therefore not considered. For inferior rectal tumors with initial sphincter involvement, the assessment of radiological response failed to provide a definitive distinction between fibrosis, edema, and tumor infiltration; these cases were consequently classified as the maximum T stage.

Surgical management involved radical resection with TME, with or without sphincter preservation, based on tumor location and characteristics. A temporary defunctioning stoma was not routinely performed and was indicated in selected cases in accordance with surgeon's experience, based on patient frailty and intraoperative findings such as significant edema, fibrosis, issues during anastomosis formation, or extensive contamination.

Complete pathological response (pCR) was defined as the absence of residual tumor cells in the surgical specimen (ypT0N0). Surgical specimens were all analyzed by the same pathological team, which had access to the patient’s clinical background and included evaluation of macroscopic and microscopic findings, TNM staging, and assessment of lymphatic, vascular, and perineural invasion, as well as Dworak tumor regression grade.

Follow-up was conducted by the oncological and surgical teams, with a focus on local or regional recurrence and distant metastases. Local recurrence was defined as relapse occurring at the site of the original surgical resection, within the bowel wall or surrounding soft tissue of the surgical bed; regional recurrence as relapse involving draining lymph nodes and/or lateral pelvic lymph nodes; and distant metastatic recurrence as secondary tumors affecting the peritoneum or other organs. Patients underwent clinical examination, tumor marker assessment (CEA and CA19-9), and abdominopelvic ultrasound every 3 months during the first postoperative year, every 6 months over the subsequent 2 years, and annually thereafter up to 5 years after surgery, in accordance with the international recommendations in effect at the time. When recurrence was suspected, CT scanning and additional targeted investigations were performed.

This is a single-center, retrospective study aiming to evaluate whether patients who achieved pCR were also clinically assessed as having cCR prior to surgery, based on MRI findings. Additional clinical and oncological variables were analyzed, including pre-treatment blood work, tumor markers, type of surgery performed, postoperative complications, local recurrence, and distant metastases.

Statistical analysis was performed using SPSS version 20 (SPSS Inc., Chicago, IL, USA). Qualitative variables are presented as absolute values and percentages, while quantitative variables are expressed as means with standard deviations (SD). The accuracy of MRI in predicting pathological response was evaluated using sensitivity (recall), calculated as the ratio of true positives to the sum of true positives and false negatives.

## 3. Results

**Figure 1:**
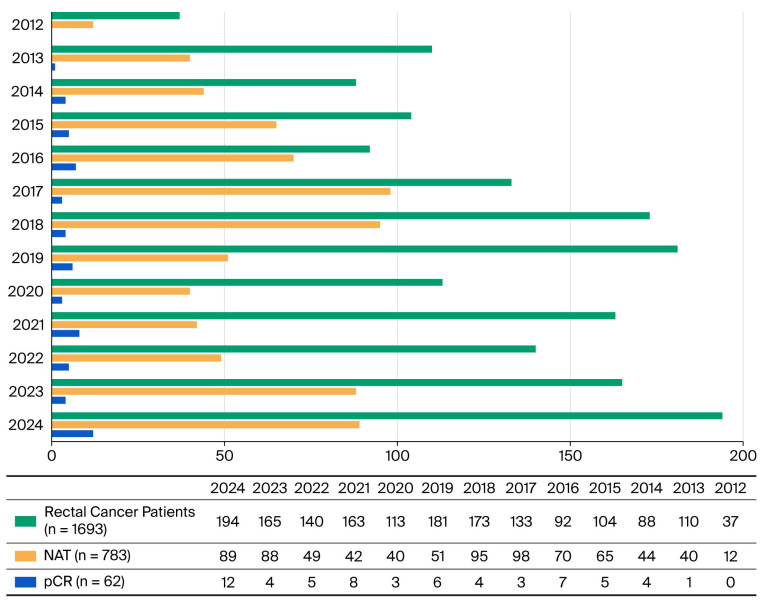
Annual distribution of patients with rectal cancer regarding NAT and pCR. NAT, neoadjuvant therapy; pCR, pathological complete response.

Out of a total of 1693 patients admitted for rectal cancer, 783 patients (46.25%) received neoadjuvant therapy followed by radical surgical treatment; 62 patients (7.92%) obtained pCR, with an annual distribution increasing up to 12 patients in 2024 (Figure 1). The average patient age was 64 years, with a minimum of 38 years and a maximum of 83 years. The male-to-female ratio was 1.21. The median follow-up time was 4 years, ranging between 1 and 12 years from the moment of radical surgical sequence. All patients included in our study underwent neoadjuvant therapy as follows: 1 patient underwent chemotherapy, having a neuroendocrine tumor type; 59 patients (95.16%) had LCRCT; 2 patients (3.22%) had SCRT; and 15 patients (24.2%) received associated neoadjuvant chemotherapy (induction or consolidation).

The baseline laboratory values, including tumor markers, are summarized in Table 1. Almost half of our cohort (45.16%) had low rectal cancer. The preferred surgical procedure was a restorative rectal resection, and depending on medical recommendations, the patient’s particularities, and the surgeon’s preference, a temporary defunctioning ileostomy or colostomy was performed. Anatomical locations, types of surgical procedures, postoperative complications, and histopathological features are presented in Table 2.

**Figure 2:**
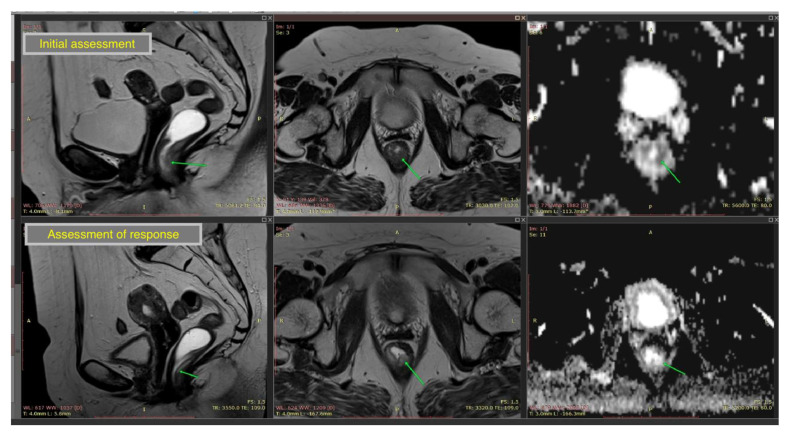
Magnetic resonance imaging initial assessment (upper three images) and response evaluation (lower three images) in an inferior rectum cancer, showing parietal thickening maintained and treatment-related changes in a patient with pathological complete response. Green arrow showing rectal tumor

A typical MRI protocol interpretation for assessing response to neoadjuvant therapy revealed rectal wall (parietal) thickening maintained but reduced in size, along with T2 hypersignal, representing either residual tumor or treatment-related changes (fibrosis, edema, inflammation): fibrosis is suggested by low signal (hypointense) and edema/inflammation by high signal (hyperintense). With tumor invasion beyond the muscularis propria, if the tumor previously extended into the subserosa or perirectal fat, post-treatment images may still show disrupted muscularis propria and fibrotic retraction bands toward the mesorectum, indicative of prior tumor spread. Locoregional lymph nodes typically decrease in size and number after effective therapy. A typical MRI image is shown in Figure 2.

Most of the patients were staged cT3 cN2b at diagnosis (Table 3), and the response to neoadjuvant treatment was assessed as cCR in 32.3% of cases regarding primary tumors and in 54.8% for lymph node involvement. Recall (sensitivity), used to measure how well the MR imaging response evaluation identified cCR, was 20/(20 + 36) = 0.36 for the tumor stage and 34/(34 + 22) = 0.6 for the lymph node stage. Although four patients presented with T4 at restaging, further workup was not performed, as it would not have changed the surgical approach, in addition to the consideration taken during the surgical act of this clinical staging. Among these locally advanced patients who showed a limited response to NAT on imagistic reevaluation, the tumor was located in the mid-upper (2 patients) and mid-lower rectum (2 patients). The final assessment and decision of surgical procedure were based on intraoperative findings (for one patient APR, two Hartmann’s procedures and one ELAPE) and mandated additional adnexectomy in one case, partial posterior colpectomy in one case, and partial prostatectomy in one case. R0 resection was supported by the pathological results, and all cases showed pCR. These cases are good examples of error in imagistic reevaluation.

Oncological outcomes are displayed in Table 4. In our cohort, there were no locoregional recurrences. Six patients from the pathological complete responders group developed liver or pulmonary metastases diagnosed after a period of 1 to 8 months after radical surgical treatment. One patient had synchronous breast cancer and needed personalized treatment.

## 4. Discussion

The number of patients treated for rectal cancer in our clinic is representative of a tertiary center specializing in oncological pathology. The number of patients who underwent NAT and who achieved pCR was similar to those in other tertiary centers, as described by Shadmanov, with a rate of 9.1% (*n* = 63) pCR over a period of 17 years [8]. Several international series reflect variable rates of complete response, ranging from 6.5 to 30% for pCR. Institutional cancer treatment protocols change in accordance with the development of international guidelines; thus, our institutional protocols have shifted over the years, from SCRT for a short period to LCRT for a longer period. Our results show a rate of response of 7.92%, increasing in recent years, probably due to the adoption of more complex neoadjuvant treatment protocols (TNT for LARC). Three trials reported approximately doubled pCR rates in patients with LARC and TNT compared with LARC and SCRT or chemoradiotherapy (CRT) with postoperative chemotherapy: the RAPIDO (28% vs. 14%), PRODIGE 23 (28% vs. 12%), and STELLAR trials (22% vs. 12%) [12,13,14,15]. In TNT, pCR is achieved at different rates depending on the protocol; for example, as described in the SPRING-01 trial, a pCR rate of 59.2% was achieved after short-course radiotherapy followed by sintilimab plus capecitabine–oxaliplatin, compared with a rate of 32.7% after short-course radiotherapy followed by capecitabine–oxaliplatin in patients with LARC [16]. We should specifically highlight that NAT in this patient cohort was not administered with the aim of inducing a cCR. Moreover, as a watch-and-wait protocol was not adopted in our MDT, none of the patients with cCR were advised to undergo watch-and-wait. All patients received counseling regarding the current practices of our clinic, mirrored by data available in the literature, and, even in cases that mandated an APR, consent was obtained for a radical approach, waiving the option for the watch and wait protocol.

Post-treatment MRI showed a complete tumor response (T0) in 20 patients (32.3%) and nodal downstaging to N0 in 34 patients (54.8%). The MRI findings described (e.g., residual fibrosis, edema, retraction) can be present despite ypT0N0—they reflect treatment effects, not active disease, but may mislead tumor response assessment. In our study, MRI provided imaging findings that indicate a limited correlation between clinical assessment of tumor response and pathological outcome. The accuracy of response assessment was lower compared to the initial staging MRI, with a sensitivity of 50.4% using only T2WI, but with a specificity of 91.2% for the detection of residual tumor [17]. In the ESCP study on 2571 patients receiving radical surgery for rectal cancer in different centers from 44 European countries (673 with NAT), 13% were understaged, and 34% were overstaged, also bringing into question the optimal time for response assessment [7,18]. In a prospective observational study evaluating tumor grade regression using MRI in rectal cancer patients for correlation with surgical specimen assessment, including 80 patients, the mrTRG-pTRG accuracy was 93.7% for complete response, with a positive predictive value of 68.7%, and a sensitivity of 88% [19].

In accordance with international guidelines, the common practice of our Unit involves TNT, even though organ preservation or a watch-and-wait approach is not planned because it improves oncologic outcomes, specifically by increasing pathological complete response rates, reducing distant metastases, and enhancing disease-free survival compared to traditional approaches. TNT involves delivering all planned chemotherapy and chemoradiation before surgery, rather than reserving some chemotherapy for the postoperative period. This sequencing maximizes compliance with systemic therapy, as postoperative chemotherapy is often poorly tolerated and incompletely delivered [20,21]. Randomized trials such as RAPIDO and PRODIGE-23 have demonstrated that TNT, regardless of subsequent surgical intent, leads to higher rates of pathological complete response and lower rates of distant metastasis than standard preoperative chemoradiation followed by surgery and adjuvant chemotherapy [12,14]. TNT is particularly indicated for patients with MRI-defined high-risk features, such as threatened mesorectal fascia or extramural venous invasion, where the risk of systemic relapse is elevated [21]. The American Society for Radiation Oncology and the Society of Surgical Oncology recognize TNT as an accepted standard of care for locally advanced rectal cancer, not solely for organ preservation but also for improving long-term oncologic outcomes [22]. Thus, TNT is appropriate for patients in whom surgery is planned, as it optimizes both local and systemic disease control, independent of organ preservation strategies.

The limited accuracy of MRI in rectal cancer restaging is mainly due to both overstaging and understaging after neoadjuvant therapy [23]. Overstaging frequently occurs because radiotherapy-induced fibrosis, inflammation, vascular proliferation, ulceration, or proctitis can mimic residual tumor, leading to overestimation of rectal wall invasion, particularly in T1–T2 tumors. Conversely, understaging results from small residual tumors being obscured by fibrotic tissue [24]. To improve accuracy, mandatory comparison of pre- and post-treatment MRI is recommended, focusing on tumor position, extension, and signal intensity. Optimized MRI protocols with high-resolution multiplanar T2-weighted imaging and mandatory diffusion-weighted imaging (DWI) can enhance differentiation between fibrosis and viable tumor. Three-dimensional MR volumetry improves the assessment of tumor downsizing and correlates with pathological stage. Functional techniques further contribute: perfusion MRI evaluates tumor vascularity and response, while ADC values from DWI can predict treatment response. Histological subtypes, especially mucinous adenocarcinoma, must be considered, as high T2 signals from mucin can lead to misinterpretation and reduced MRI accuracy [25].

The practice in our service is of pelvic MRI-based restaging and is based on resource limitations, supported by data from both prospective and retrospective studies that demonstrate that the incidence of new distant metastases detected by chest and abdominal CT after NAT is low (approximately 3%) and the impact on treatment strategy is minimal [26]. However, we do acknowledge this aspect as one limitation of this study, as data regarding metastatic disease at the time of surgery may be affected.

Our cohort had no locoregional recurrence. Several studies have reported local recurrence rates after pathological complete response (pCR) in rectal cancer, ranging between 1% and 5%. In a study published in 2017 with a cohort of 195 patients who achieved pCR, regional recurrence occurred in 1.5% of patients with lower-rectum tumors and pre-treatment lateral LN metastasis. In the same study, the distant recurrence rate was 7.7% [27]. In 2023, a multicenter observational study was published with 32 different Spanish centers participating, involving 815 rectal cancer patients with pCR. The rate of local recurrences was 1.8%, and only 20% of them had lateral localization [28].

In our study, we observed a distant recurrence rate of 9.67% among patients who achieved a pCR following neoadjuvant chemoradiotherapy and surgery. This included four cases of single liver metastasis that were surgically excised within the first 8 months postoperatively, as well as one case each of unresectable bilobar liver metastasis and pulmonary metastasis, both of which were managed with adjuvant chemotherapy. These findings align with those of prior studies that have reported distant recurrence rates of approximately 7–15% in patients achieving pCR. For instance, Maas et al. (2010) performed a pooled analysis of 484 pCR patients and found a 5-year distant recurrence rate of approximately 8%, highlighting that while pCR is associated with favorable outcomes, systemic relapse remains a clinical concern [29]. These findings are consistent with the recent literature. The 2023 multicenter study from the Spanish Rectal Cancer Project reported a 6.4% incidence of distant metastasis at a median follow-up of 73.4 months. Notably, abdominoperineal excision and elevated carcinoembryonic antigen (CEA) levels were identified as independent risk factors for distant recurrence [28].

An evaluation of tumor markers revealed that CEA and CA19-9 levels were generally within normal ranges across our cohort. However, one patient with metachronous colon cancer exhibited elevated CEA levels. Additionally, two patients who later developed liver metastases showed slightly elevated CA19-9 levels (25 and 47 U/mL, respectively).

An analysis of the International Watch & Wait Database, which focused on the risk of distant metastasis in patients with cCR managed by W&W after neoadjuvant therapy, found that the 5-year distant metastasis rate was 8.1% [10]—the same as reported in a meta-analysis published by Dattani et al. in 2018 [30]. Importantly, the occurrence of local regrowth was associated with a higher risk of subsequent distant metastasis. Moreover, Habr-Gama et al. and other proponents of nonoperative management strategies have also reported distant recurrence in 10–15% of patients with a sustained clinical complete response (cCR), often within the first two years of follow-up [31].

Our rate of liver metastases (8.06%) is noteworthy, especially given that the majority were solitary and resectable, underscoring the importance of vigilant postoperative surveillance and the potential for curative salvage strategies. The low incidence of pulmonary metastasis (1.61%; one patient) is consistent with previously published patterns of metastatic spread, where the liver remains the most common site [32].

A recent systematic review and meta-analysis conducted by Sugumar et al., investigating the trial-level association between pCR and survival in rectal cancer patients, found no correlation with DFS and OS, concluding that it is not a surrogate endpoint of long-term outcomes in matters of neoadjuvant treatment, including TNT and systemic disease [33]. While pCR may reduce the risk of local recurrence—potentially compensating for imperfections in surgical technique—it does not necessarily predict systemic disease control [34]. This is because systemic dissemination often occurs before the initiation of treatment; therefore, a complete local response does not guarantee the absence of micrometastatic disease.

This study has several limitations, primarily due to its retrospective design. The main focus was on patients achieving pCR, without considering NAT or clinical assessment as selection criteria. We relied solely on available patient charts and MRI interpretations, in some cases without access to the original images, so individual comparison of images was not possible; however, as mentioned above, the MRI interpretation followed certain protocols, reducing the risk of bias. Although the MRI technology and interpretation criteria evolved over the study period, potentially affecting the consistency of clinical staging, we reviewed images and imaging descriptions with TNM staging according to the 8th edition of the American Joint Committee on Cancer (AJCC) TNM classification.

We were not able to definitively characterize each tumor, considering the risk factors for local recurrences or progressive disease. Furthermore, the absence of more consistent survival data is one of the main limitations of this study, which may be addressed through future prospective approaches to the gathering of data.

## 5. Conclusions

While MRI provides valuable preoperative information, its accuracy in predicting pCR remains limited. These findings support the need for improved imaging or biomarker-based tools to guide nonoperative strategies in rectal cancer management.

Achieving pCR is a favorable short-term prognostic biomarker, but it does not eliminate the risk of distant metastasis. Therefore, continued surveillance and individualized management strategies remain essential to optimize long-term outcomes in rectal cancer patients.

## Figures and Tables

**Table 1:** Baseline laboratory findings.

Descriptive Statistics	N	Minimum	Maximum	Mean	Std. Deviation
Serum Albumin	41	3.80	5.53	4.6002	0.35351
Hemoglobin	58	9.00	17.50	13.3397	1.67237
Leucocytes	58	2630.00	9300.00	5333.7931	1488.39703
Neutrophils	58	710.00	7450.00	3702.4138	1285.74275
Lymphocytes	58	430.00	4900.00	1154.8276	718.13169
Platelets	58	103,000.00	468,000.00	229,189.6552	64,651.41534
CEA	54	0.00	5.56	1.5876	1.14983
CA19.9	55	0.60	2660.00	59.6335	357.50511
Valid N (listwise)	41				

**Table 2:** Anatomical locations, types of surgical procedures, postoperative complications, and histopathology.

Patient’s Characteristics		Value (Number of Patients, Percentage)	
Anatomical location			
Inferior rectum		28 (45.16%)	
Mid–lower rectum		13 (20.96%)	
Mid rectum		17 (27.41%)	
Mid–upper rectum		6 (9.68%)	
Type of surgical procedure			
Anterior rectal resection	Anterior rectal resection	33 (53.22%)	2 (3.22%)
	Low anterior rectal resection		6 (9.67%)
	Ultralow anterior rectal resection		25 (40.32%)
Hartmann’s procedure		4 (6.45%)	
Extralevator abdominoperineal excision		25 (40.32%)	
Temporary defunctioning stoma			
Temporary ileostomy		11 (17.74%)	
Temporary colostomy		2 (3.22%)	
Postoperative complications			
Anastomotic leak		3 (4.83%)	
Parastomal cellulitis		1 (1.61%)	
Clostridium difficile enterocolitis		3 (4.83%)	
Urinary tract infection		1 (1.61%)	
Respiratory tract infection		1 (1.61%)	
Histopathology			
Adenocarcinoma → 2 mucinous type		59 (95.1%)	
Squamous-cell carcinoma		2 (3.2%)	
Neuroendocrine tumor		1 (1.6%)	

**Table 3:** Clinical staging considering tumor and lymph node stage before (cT and cN) and after neoadjuvant treatment (ycT and ycN).

**Tumor Stage**	**0**	**1**	**2**	**3**	**4A**	**4B**
cT			5 pts (8.1%)	45 pts (72.6%)	5 pts (8.1%)	7 pts (11.3%)
ycT	20 pts (32.3%)	8 pts (12.9%)	10 pts (16.1%)	14 pts (22.6%)	2 pts (3.2%)	2 pts (3.2%)
**Lymph Node Stage**	**0**	**1A**	**1B**	**2A**	**2B**	
cN	7 pts (11.3%)	2 pts (3.2%)	14 pts (22.6%)	14 pts (22.6%)	25 pts (40.3%)	
ycN	34 pts (54.8%)	5 pts (8.1%)	13 pts (21%)		4 pts (6.5%)	

**Table 4:** Oncological outcomes.

Event	Number of Patients, Time of Occurrence
Locoregional recurrence	0 pts
Single liver metastasis surgically excised	4 pts (6.5%) 2–8 months after TME
Multiple bilobar liver metastasis	1 pt (1.61%) 5 months after TME
Pulmonary metastasis	1 pt (1.61%) 1 month after TME
Synchronous breast cancer	1 pt (1.61%) Hormonotherapy, breast adjuvant radiotherapy

## Data Availability

The data presented in this study are available on request from the corresponding author due to privacy and legal particularities.

## Abbreviations

The following abbreviations are used in this manuscript:

MRI	Magnetic Resonance Imaging
pCR	Pathological Complete Response
cT	Clinical Tumor Stage
cN	Clinical Nodal Stage
ycT	Post-treatment Clinical Tumor Stage
ycN	Post-treatment Clinical Nodal Stage
TNM	Tumor, Node, Metastasis classification
AJCC	American Joint Committee on Cancer
NAT	Neoadjuvant Treatment
cCR	Clinical Complete Response
mrTRG	Magnetic Resonance Tumor Regression Grade
pTRG	Pathologic Tumor Regression Grade
W&W	Watch and Wait
CT	Computed Tomography
LCRT	Long-Course Radiotherapy
LCRCT	Long-Course Chemoradiotherapy
SCRT	Short-Course Radiotherapy
5-FU	5-Fluorouracil
TME	Total Mesorectal Excision
MDT	Multidisciplinary Team
SD	Standard Deviation
T2WI	T2-Weighted Imaging
LN	Lymph Node
LARC	Locally Advanced Rectal Cancer
TNT	Total Neoadjuvant Therapy
CEA	Carcinoembryonic Antigen
CA19-9	Carbohydrate Antigen 19-9
DFS	Disease-Free Survival
OS	Overall Survival
